# The Impact of Digital Leadership on Employee Resilience: The Mediating Roles of Work Gamification and Workplace Mindfulness and the Moderating Role of AI Anxiety

**DOI:** 10.3390/bs16050644

**Published:** 2026-04-25

**Authors:** Yanshu Ji, Xiaoyi Wang, Lijun Xia, Huabin Wu

**Affiliations:** 1 School of Business, Nanjing University, Hankou Road 22, Nanjing 210093, China; yanshuji@smail.nju.edu.cn; 2 School of Entrepreneurship, Zhejiang University of Finance and Economics Dongfang College, No. 2 Yangshan Road, Haining 314408, China; 2420110221@zufedfc.edu.cn

**Keywords:** digital leadership, employee resilience, work gamification, workplace mindfulness, AI anxiety

## Abstract

Despite the rapid penetration of digital technologies into the workplace, many enterprises are undergoing digital transformation, making safeguarding employees’ occupational health increasingly important. Drawing on social cognition and the conservation of resources theories, this study developed a moderated mediation model to explore the relationship between digital leadership and employee resilience, as well as the mediating roles of work gamification and workplace mindfulness and the moderating role of AI anxiety. Survey data from 293 employees revealed that digital leadership significantly and positively predicted employee resilience. Both work gamification and workplace mindfulness mediate the relationship between digital leadership and employee resilience. AI anxiety positively regulated the positive relationship between workplace mindfulness and employee resilience but did not significantly moderate the indirect pathway through work gamification. In addition, AI anxiety negatively regulates the direct positive effect of digital leadership on employee resilience. These findings clarify the mechanisms and boundary conditions through which digital leadership promotes employee resilience in digitally transformed workplaces. This study also offers practical implications for organizations seeking to protect employee well-being and reduce burnout during digital transformation.

## 1. Introduction

In the era of the digital economy, enterprises are continuously introducing advanced digital technologies such as big data and artificial intelligence into relevant fields to keep pace with the times and achieve sustained progress and development. According to the global survey report *The State of AI in 2025* released by McKinsey in 2025, 88% of the surveyed enterprises have routinely adopted artificial intelligence technology in at least one business. Forty-two percent of the enterprises plan to improve their employees’ AI skills by 2025. However, the mere introduction of technology is often accompanied by substantial organizational risk. According to the survey data from McKinsey (2025), 32% of the respondents predict that the total number of employees in enterprises will decrease by more than 3% because of AI. If this is the case, it can be seen that employees can feel the strong impact of AI technology. If enterprises neglect employees’ psychological adaptability and behavioral transformation during the process of digitalization, this can easily lead to technostress, job burnout, and even high turnover rates, resulting in serious economic and efficiency losses and becoming a major reason for the failure of digital transformation in many firms (Trenerry et al., 2021). Therefore, against this high-risk backdrop of digitalization, it is particularly important to focus on and enhance employees’ psychological and behavioral stability when facing technological shocks. This study focuses on enterprises during the period of digital transformation and explores the relationship between digital leadership and employee resilience (Arranz Lahuerta et al., 2026).

Under the severe challenges of digital transformation described above, the core issue confronting enterprises is how to effectively mobilize and utilize managers’ digital resources and capabilities, namely, digital leadership, to substantially enhance employee resilience. Employee resilience is a core driving force that enables enterprises to achieve effective transformation and development through digital technologies (Luan et al., 2025). As the foundation of the enterprise, employees who can adapt to the rapidly changing technological environment can effectively apply digital technologies in key areas, thereby addressing the practical difficulty of technology implementation within firms (Näswall et al., 2019; Prayag & Dassanayake, 2023). This study aims to answer the following question: can digital leadership effectively enhance employee resilience through specific work mechanisms and psychological conditions?

Employee resilience in the workplace is a core issue in the field of organizational behavior. In terms of measuring employee resilience, there has been a shift from the initial incorrect use of psychological resilience scales to the later accurate application of employee resilience scales. With respect to how to improve employee resilience, the literature has explored this issue from two main perspectives: individual factors and environmental factors. In terms of individual factors, researchers have confirmed from an internal perspective that individual differences such as employees’ emotions and proactive personalities positively influence their own resilience (Zhu & Li, 2021; Luan et al., 2025; Wu et al., 2025a), and researchers have paid attention to the spillover effects of leaders’ individual characteristics, such as the level of spiritual leadership (G. Li & Hu, 2025; Wu et al., 2025b). In terms of environmental factors, scholars have proposed that internal organizational environments such as high-performance work systems can effectively strengthen employee resilience and benefit both individuals and organizations (Cooke et al., 2016; Jung & Kim, 2026).

Although existing research has provided a solid theoretical foundation for understanding employee resilience, several clear limitations remain. Most prior studies have largely overlooked the antecedents of employee resilience within the specific contemporary context of the digital technology environment, and they have failed to systematically link leaders’ ability to perceive and utilize digital resources, namely, digital leadership, with employee resilience. Few studies have combined digital leadership with employee resilience for the following reasons: First, the recent intensification of digitalization in enterprises has led to digital capabilities not only existing in large enterprises but also being valued by small and medium-sized enterprises, laying a solid foundation for further research by scholars. Second, owing to the profit-oriented nature of most enterprises, the existing research is mostly limited to the organizational level. The primary purpose of introducing digital leadership is to drive the correct transformation of enterprises and consolidate their market position, which makes it easier to overlook the micro-level of individual employees. However, the successful development of an enterprise cannot be achieved without the efforts of its employees. If the relationship between digital leadership and employee resilience is ignored, when digitalization impacts employee resilience, leaders will lack a useful theoretical framework for intervention. In addition, during the process of digital innovation, firms are highly susceptible to the emergence of the “digital transformation paradox” (Karhade & Dong, 2021), whereby digital technologies may challenge managers’ authority and trigger resistance to technology, and employees may experience severe anxiety (Anthony, 2018; Christodoulou et al., 2022). This not only undermines employees’ psychological defences but can also lead to organization-level digital resistance, trapping firms on the “dark side” of digitalization (Marsh et al., 2022). Therefore, in the context of a working environment where AI technology is being increasingly utilized, exploring how digital leadership can effectively enhance employee resilience and investigating the role that AI anxiety plays in this process are highly important.

To address these shortcomings, this study aimed to systematically reveal the mechanism and boundary conditions through which digital leadership influences employee resilience on the basis of the unidirectional transmission path of “environment → individual → behavior” in social cognitive theory while also incorporating conservation of resources theory. As a novel work system, work gamification can stimulate employees’ positive emotions and help them accumulate psychological resources to adapt to significant environmental change (Decha, 2025); Taşkan Burcu et al. reported that workplace mindfulness can mediate the relationship between uncertainty and adaptive performance, effectively linking the environment and behavior (Taşkan et al., 2025). Grounded in the environment, people, and behavior, this study incorporates work gamification as the institutional environment and workplace mindfulness as employees’ individual cognition as a mediating variable and introduces AI anxiety as a moderating variable into the theoretical model. The scope of this study is limited to self-reported perceptual data collected from employees in Chinese enterprises. The highly condensed conclusion of this thesis is that digital leadership not only positively predicts employee resilience but also exerts indirect positive effects by improving work gamification and workplace mindfulness. According to the Conservation of Resources theory, resources are the driving force of human behavior and psychological states. When resources are at risk of being lost, people experience stress and tension. AI anxiety is a negative emotion that employees feel when they are worried about losing their jobs because of the impact of AI. On the one hand, employees with high levels of AI anxiety feel threatened when facing digital leadership promoted by the enterprise, thus being immersed in tension and finding it difficult to engage in learning digital technologies. However, when the level of AI anxiety is high, employees mobilize new resources to avoid losing their own resources and follow the organization to mobilize mindfulness resources to enhance their resilience. Employee resilience is the result of resource mobilization. Therefore, AI anxiety acts as a double-edged sword: it weakens the direct empowering effect of digital leadership but to some extent stimulates employees to activate mindful cognition, thereby strengthening the indirect pathway to resilience.

The innovation points of this research lie in the following aspects. First, it expands the research scope of digital leadership from the macro level of the organization to the micro level of employee resilience. Second, it combines social cognitive theory with resource conservation theory to construct a model and explain the differential moderating effect of AI anxiety. Third, it broadens the boundary conditions of employee resilience by incorporating AI anxiety as a moderating variable into the model, adapting to changes in the work environment, and helping leaders enhance employee resilience through their influence or internal organizational arrangements.

The remainder of this paper is organized as follows. The first section presents the literature review and research hypotheses, in which the concepts of the relevant variables are systematically reviewed, and the underlying mechanisms are theoretically derived. The second section describes the research design, including the sample collection process and measurement instruments for the variables. The third section reports the data analysis and results, in which the theoretical model is tested empirically. The fourth section provides conclusions and policy recommendations and discusses the theoretical contributions, managerial implications, and future directions of the study.

## 2. Literature Review and Research Hypotheses

### 2.1. Technology Resource Factor: Digital Leadership

In the digital age, the changes in and development of leadership have attracted considerable attention from scholars in recent years. Kane et al. were the first to study how digital disruption has transformed business and leadership and proposed new requirements for traditional leadership skills (Kane et al., 2019). Contreras et al. proposed that in the special environment of the pandemic, effective leadership is needed to guide employees in using electronic technology to enhance the adaptability of enterprises (Contreras et al., 2020). As digital technology gradually shifts from a technical support role to a key driver of organizational change, the concept and application of digital leadership have also been refined. Digital leadership can be broadly defined as leaders’ ability to understand digital technologies and risk management, as well as their ability to utilize digital technology-based work tools (Imran et al., 2025; Van Wart et al., 2019). More specifically, digital leadership encompasses leaders’ ability to leverage existing digital resources to influence employees, make sound decisions in response to events within digital contexts, demonstrate empathy toward employees in technology-mediated work settings, and enhance employees’ job performance through effective leadership behaviors (Avolio et al., 2014; Roman et al., 2019). Taken together, these interrelated competencies provide a comprehensive basis for assessing whether organizational leaders genuinely possess digital leadership and whether they can facilitate organizational innovation and development through digitally oriented strategies. Existing measurement tools for digital leadership are developed mainly on the basis of Western scenarios. Some use unidimensional scales for measurement, while others use multidimensional scales. Roman et al. divided digital leadership into six dimensions: communication, social, flexibility, team, and trust (Roman et al., 2019).

Previous research has focused primarily on the effects of digital leadership on firm-level outcomes such as organizational resilience and performance (Mollah et al., 2023; Ye, 2025). However, some studies have shown that when leaders introduce new technologies into the workplace and guide employees in their use, they can encourage job crafting and motivate employees to improve their digital competence (Shin et al., 2023; Ye, 2025). In doing so, leaders help employees move beyond entrenched work routines and adapt better to current job demands. Leaders with strong digital capabilities are more likely to introduce relevant digital technologies in a timely manner and provide appropriate guidance for their use, thereby reducing repetitive work and enhancing employees’ knowledge and skills. Consequently, employees may experience greater job satisfaction, stronger work focus, and broader capability development. On the basis of the above reasoning, this study proposes the following hypothesis:

**Hypothesis 1** **(H1).** *Digital leadership is positively related to employee resilience.*

In addition, digital technologies enable organizations to introduce gamified work elements and related digital platforms, which may jointly contribute to the development of employee capabilities. From the perspective of social cognitive theory, digital leadership may influence employee resilience not only through organizational resources but also by shaping the internal organizational environment and employees’ cognitive states. Therefore, beyond the resource-based perspective, this study further examines two specific pathways through which digital leadership affects employee resilience: an institutional pathway through the promotion of work gamification and a cognitive pathway through the enhancement of workplace mindfulness.

### 2.2. Institutional Environment Factor: The Mediating Role of Work Gamification

Pelling defined gamification as the application of game elements to the learning process in any field. Deterding et al. proposed that gamification is the use of game elements in non-gamified domains. Subsequently, gamification has been widely applied in various scenarios (Deterding et al., 2011), laying the foundation for research on work gamification. Work gamification refers to a management practice that integrates the hedonic features of games with the instrumental nature of work (Hamari, 2017; S. Li et al., 2025). As a form of organizational management, work gamification can be applied to both organizational and self-regulation, enabling employees to actively acquire performance-related information and improve task completion in a more engaging and enjoyable way (Cardador et al., 2017; Gerdenitsch et al., 2020). Work gamification typically includes elements such as leaderboards, personal progress indicators, and virtual identities (S. Li et al., 2025), and the degree of gamification within an enterprise’s internal work is usually determined by the number of gamification elements.

Integrating game elements into work systems requires the integration of multiple fields, such as computer science and game design, and is dependent not only on relevant digital technologies, such as gamification platforms and software but also on leaders’ ability to guide employees in using such technologies within gamified management processes. Digital leadership reflects leaders’ ability to utilize digital platforms, as well as their sensitivity to and understanding of digital technologies (Imran et al., 2025). Existing studies indirectly suggest that leaders with stronger and more comprehensive digital leadership are more likely to accept new technologies, introduce appropriate gamification platforms, and encourage employees to engage with gamified work systems, thereby increasing the level of work gamification in the organization. On the basis of the above reasoning, this study proposes the following hypothesis:

**Hypothesis 2** **(H2).** *Digital leadership is positively related to work gamification.*

Work gamification can make repetitive tasks more interesting, thereby increasing employees’ motivation to complete their tasks and providing them with positive feedback, which helps regulate stress levels (Ruggiu et al., 2022). In gamified work settings, organizations can, on the one hand, make employees’ contributions more visible through tools such as point systems and leaderboards, thereby enhancing employees’ sense of self-worth and promoting self-driven development in fragmented work contexts (Morschheuser & Hamari, 2019). In contrast, according to social cognitive theory, environmental factors can directly influence behavioral outcomes. By establishing a well-designed gamified work system, organizations can create a supportive environment for growth and constructive competition, thereby strengthening employees’ psychological endurance, learning, and adaptive capacities.

From the perspective of the organizational technological environment, digital leadership may indirectly influence employee resilience through gamification. Prior research has shown that digital technology development facilitates the implementation of gamified human resource management practices (S. Li et al., 2025). On the basis of their understanding of organizational conditions and awareness of digital technologies, managers can introduce relevant tools to support gamified management. Because work gamification highlights the importance of intrinsic motivation in the work process (Huang & Zhou, 2025), its implementation may enhance employees’ intrinsic motivation and participation, sustain positive psychological states, and strengthen learning capability and resilience. Accordingly, this study proposes the following hypothesis:

**Hypothesis 3** **(H3).** *Work gamification mediates the relationship between digital leadership and employee resilience such that digital leadership enhances employee resilience by increasing work gamification.*

### 2.3. Employee Cognitive Factors: The Mediating Role of Workplace Mindfulness

With the advancement of digital technologies, organizations increasingly expect employees to maintain high levels of efficiency to improve organizational performance. However, excessive work demands may intensify employees’ anxiety and undermine their attentional focus. Kabat-Zinn argued that one effective way to address this challenge is to enhance employees’ mindfulness (Kabat-Zinn, 2003), which has gradually been recognized as an important response to digitalized work contexts (Mrazek et al., 2019). Originating from an ancient concept in Eastern Buddhism, mindfulness has been incorporated into organizational research and is generally understood in workplace settings as involving four key elements: sustained attention, awareness, present-centered focus, and non-judgmental acceptance (Brown et al., 2007; Zhang et al., 2017). Workplace mindfulness refers to an employee’s active tendency to direct consciousness and attention to present work tasks and the immediate work environment in a non-judgmental manner (Kabat-Zinn, 2003). In terms of measurement, early measures of workplace mindfulness mostly utilized single-dimensional scales. In recent years, Zheng et al. developed a three-dimensional scale for workplace mindfulness that is based on work scenarios, which has also made the measurement results more accurate.

Existing research suggests that employees can improve their mindfulness through formal practices, such as meditation training. With their accessibility and convenience, digital mobile applications allow employees to engage in mindfulness practices anytime and anywhere. Studies further indicate that when digital technologies or mobile applications are introduced or designed by organizational leaders, they can support employees’ psychological self-monitoring, improve mindfulness, and reduce workplace stress (Wrede et al., 2025). Leaders with strong digital leadership are more capable of introducing and systematically optimizing digital mindfulness tools in accordance with organizational needs, thereby enabling them to benefit from digital resources and improve their mindfulness through digital mindfulness interventions. Accordingly, digital leadership is likely to be closely associated with workplace mindfulness. Therefore, this study proposes the following hypothesis:

**Hypothesis 4** **(H4).** *Digital leadership is positively related to workplace mindfulness.*

Mindfulness is a positive individual characteristic that helps employees improve their cognitive functioning, strengthen their emotional regulation, and develop their adaptive capacity (Cheng et al., 2023). Previous studies have shown that mindfulness interventions have a significant moderate effect on reducing perceived stress (Mrazek et al., 2019), suggesting that organizations can effectively alleviate employees’ work stress by enhancing their level of mindfulness. Employee resilience refers to the ability of employees to adapt to, cope with, and grow in the face of constantly changing environments (Tonkin et al., 2018). Enterprises can positively influence employees’ resilience by enhancing workplace mindfulness to mobilize their psychological resources. From the perspective of social cognitive theory, individuals’ cognition develops in response to environmental changes and subsequently shapes distinctive behavioral outcomes (Lee & Tseng, 2024). As a form of resource-based leadership, digital leadership introduces and mobilizes digital resources that can be used to enhance employees’ mindfulness. Through mindfulness-related training and digital support, employees can strengthen their cognitive capabilities, which, in turn, can improve their behavioral adaptability and resilience. On the basis of this reasoning, this study proposes the following hypothesis:

**Hypothesis 5** **(H5).** *Workplace mindfulness mediates the relationship between digital leadership and employee resilience such that digital leadership enhances employee resilience by increasing employees’ level of mindfulness in the workplace.*

### 2.4. The Moderating Role of AI Anxiety

Today, many organizations are choosing to integrate artificial intelligence (AI) into their operations. Although this trend creates new opportunities and improves organizational productivity, it also gives rise to negative emotional reactions among employees. Li and Huang hold that AI anxiety is the fear and concern of individuals about losing control because of the rapid development of AI technology (J. Li & Huang, 2020). Frenkenberg and Hochman proposed that AI anxiety consists of two major dimensions: anticipatory anxiety and annihilation anxiety (Frenkenberg & Hochman, 2025). Anticipatory anxiety refers to employees’ concerns that they may be unable to master AI technologies and therefore fail to complete their work in a timely manner; more seriously, they may fear that their jobs will eventually be replaced by other workers or by AI itself. Annihilation anxiety, by contrast, refers to anxiety over the potential loss of personal autonomy. For organizational employees, AI anxiety manifests primarily as anticipatory anxiety. When surrounding coworkers demonstrate superior technological competence, employees may experience anxiety because they perceive themselves to be under threat (J. Li & Huang, 2020). Accordingly, some studies suggest that the root of AI anxiety lies in broader forms of technology anxiety, as employees may develop negative emotions when they are required to learn and master complex AI algorithms (Chai & Sha, 2025). When AI anxiety is measured, multi-dimensional scales are generally used, covering multiple dimensions to comprehensively measure expected anxiety and annihilation anxiety. Li and Huang first comprehensively constructed a theoretical framework for AI anxiety, dividing it into eight core dimensions: privacy invasion anxiety, algorithm bias anxiety, job replacement anxiety, and technology learning anxiety (J. Li & Huang, 2020). Wang et al. further developed a universal AI anxiety scale (AIAS), making subsequent measurements more applicable (Wang et al., 2022).

In highly dynamic environments, employees may experience AI anxiety, leading them to worry about being replaced and consequently developing negative attitudes toward organizational AI systems. Existing research has shown that anxious individuals tend to rely more heavily on resources that provide long-term benefits (Chen et al., 2025). Moreover, gamified work processes may enable employees to gain positive and even transcendent psychological experiences through game elements (S. Li et al., 2025), thereby helping to alleviate the anxiety associated with digital technologies and strengthen employee resilience from a psychological perspective. In this sense, gamification can serve as an organizational resource from which employees can derive long-term benefits. Accordingly, when employees experience higher levels of AI anxiety, they may be more likely to engage in gamified work systems to divert or reduce their AI-related anxiety. In contrast, when AI anxiety is low, employees may perceive gamification as an additional source of pressure, which may reduce their willingness to participate and weaken its developmental benefits. Similarly, workplace mindfulness refers to a work state characterized by present-centered attention and non-judgmental awareness. From the perspective of social cognition and conservation of resources theories, it can be understood as both a cognitive capability and a psychological resource. When AI anxiety is low, employees may not feel threatened in terms of work resources and feel little need to mobilize their cognitive resources. However, when AI anxiety is high, employees are more likely to perceive environmental instability and, in response, start looking for available resources and activate their mindfulness capacity to strengthen their resilience. On the basis of the above reasoning, this study proposes the following hypotheses:

**Hypothesis 6a** **(H6a).** *AI anxiety positively moderates the relationship between work gamification and employee resilience such that the positive effect of work gamification on employee resilience is stronger when employees experience greater AI anxiety.*

**Hypothesis 6b** **(H6b).** *AI anxiety positively moderates the relationship between workplace mindfulness and employee resilience such that the positive effect of workplace mindfulness on employee resilience is stronger when employees’ AI anxiety is higher.*

Building on this logic, this study further proposes moderated mediation hypotheses, suggesting that the two indirect pathways through which digital leadership influences employee resilience—work gamification and workplace mindfulness—are contingent on the level of AI anxiety. When AI anxiety is high, organizational leaders may be more motivated to introduce gamified elements that fit the organization’s developmental needs and place greater emphasis on mindfulness training for employees. Simultaneously, higher AI anxiety may encourage employees to participate more actively in gamified work systems and improve their own level of mindfulness. Therefore, this study proposes the following hypotheses:

**Hypothesis 7a** **(H7a).** *AI anxiety positively moderates the mediating effect of work gamification on the relationship between digital leadership and employee resilience such that the indirect effect of digital leadership on employee resilience through work gamification is stronger when AI anxiety is greater.*

**Hypothesis 7b** **(H7b).** *AI anxiety positively moderates the mediating effect of workplace mindfulness on the relationship between digital leadership and employee resilience such that the indirect effect of digital leadership on employee resilience through workplace mindfulness is stronger when AI anxiety is higher.*

In addition to moderating the two indirect pathways, this study argues that AI anxiety moderates the direct effect of digital leadership on employee resilience. According to conservation of resources theory, employees with high levels of AI anxiety may perceive digital technologies as threats rather than as resources, thereby triggering defensive responses and reinforcing their reliance on established work routines. Consequently, they may be less willing or able to effectively use the digital tools provided by leaders, which may hinder their progress in mastering digital technologies. Furthermore, when employees are heavily affected by AI anxiety, the direct empowering effect of digital leadership may be less effective in enhancing employee resilience. Under such circumstances, organizations may need to rely more heavily on alternative mechanisms, such as improving the level of work gamification and enhancing employees’ mindfulness, to strengthen employee resilience. Accordingly, this study proposes the following hypothesis:

**Hypothesis 8** **(H8).** *AI anxiety negatively moderates the relationship between digital leadership and employee resilience such that the positive effect of digital leadership on employee resilience is weaker when employees’ AI anxiety is high.*

This study incorporates both work gamification and workplace mindfulness simultaneously into the mediating path without establishing a chain mediation through which digital leadership influences employee resilience via work gamification and workplace mindfulness. The main reasons are as follows: First, the data collected in this study are cross-sectional and cannot effectively capture the chain mediating relationship between gamification and mindfulness. Chain mediation requires a clear temporal sequence, but it is difficult to observe how employees’ level of mindfulness changes during the process of implementing and improving work gamification using questionnaire data. Second, from the perspective of resources, work gamification is from the enterprise resource perspective and provides work guidance for employees, whereas workplace mindfulness is from the individual resource perspective and provides psychological guidance for employees. This is a parallel relationship, and it reflects that the environment and individuals can influence behavior.

**Figure 1:**
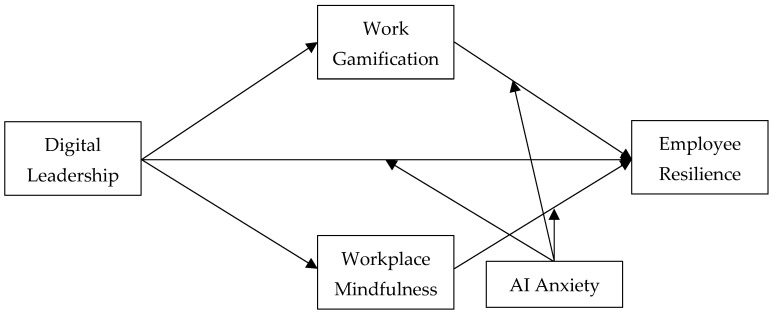
Theoretical model.

In summary, this study proposes a moderated partial mediation model (Figure 1).

## 3. Materials and Methods

### 3.1. Research Sample and Data Collection

This study adopted a cross-sectional design, and all variables were collected at the same time point through the employees filling out questionnaires. The questionnaire emphasized that the data filled in this time would be used for academic research and would be anonymous. There was no right or wrong answer for the filling results. The key variables included digital leadership, work gamification, workplace mindfulness, employee resilience, AI anxiety, and several control variables. The sample was primarily drawn from employees working in various types of enterprises. Specifically, the data collection covered employees from state-owned enterprises during the period of digital transformation, private enterprises, and foreign-invested enterprises, as well as organizations located in different regions, to ensure sample diversity and enhance the generalizability of the findings. To improve the standardization and quality of questionnaire completion, the survey was distributed in multiple small batches at different points in time. In this study, participants were also recruited through two channels. First, the questionnaire link was distributed to the internal staff of enterprises via the human resources department, allowing employees to fill it out themselves. Second, the questionnaire was distributed through online platforms, with the premise of the digital transformation background, to involve a wider range of workers. The distribution of the questionnaire was not limited to any specific region and covered employees from multiple provinces and cities. In total, 400 questionnaires were distributed, of which 337 were returned. After 44 invalid or low-quality questionnaires were excluded, a final sample of 293 valid responses was retained, yielding an effective response rate of 73.25%. Among the respondents, 53.6% were male and 46.4% were female. Most participants were between 30 and 45 years old. In terms of educational background, 48.8% held a bachelor’s degree. Most respondents worked in enterprises with 51 to 500 employees. The sample also covers a wide range of enterprise ownership types, and 42.3% of the respondents were from firms located in eastern China. The detailed sample characteristics are presented in Table 1.

### 3.2. Variable Measurement

All the variables were measured using well-established scales from the domestic and international literature. Foreign-language scales were translated into Chinese using a translation–back-translation procedure. All the items were rated on a five-point Likert scale ranging from 1 (strongly disagree) to 5 (strongly agree).

Digital leadership (DL) was measured using a six-item scale developed by Zeike et al. (2019) and completed by employees. Representative items include “My leader finds it interesting to use digital tools” and “My leader would describe themselves as a digital expert.” The Cronbach’s α for this scale was 0.931.

Work gamification (WG) was measured using the twelve-item scale developed by S. Li et al. (2025), which assesses the degree of work gamification through three dimensions: achievement, social interaction, and immersion. The digital elements of this scale were adapted to specific items for the present study. Representative items include “The organization requires us to use avatars/virtual identities” and “The organization provides us with customized/personalized content.” The Cronbach’s α was 0.881.

Workplace mindfulness (WM) was measured using the eighteen-item scale developed by Zheng et al. (2022), which assesses workplace mindfulness across three dimensions: awareness, attention, and acceptance. The representative items include “I focus my attention on my current work” and “At work, I do not judge my thoughts as good or bad.” The Cronbach’s α was 0.926.

AI anxiety (AIA) was measured in two dimensions—AI learning anxiety and job replacement anxiety—using the multidimensional scale developed by J. Li and Huang (2020). The results of the exploratory factor analysis indicated that the six items of AI anxiety extracted one common factor (KMO = 0.877, cumulative variance explained rate = 63.974%), with item loadings ranging from 0.687 to 0.866, suggesting that it is feasible to treat AI anxiety as a whole. Meanwhile, the limitations section discusses the theoretical differences between the two dimensions. Representative items include “AI technology is updated too quickly for me to keep up” and “I worry that AI will replace many people’s jobs”. The Cronbach’s α was 0.886.

Employee resilience (ER) was measured using a nine-item scale developed by Näswall et al. (2019). Representative items include “I effectively manage crises at work” and “I effectively respond to feedback at work, even criticism.” The Cronbach’s α was 0.948.

## 4. Results

### 4.1. Confirmatory Factor Analysis

Confirmatory factor analysis (CFA) was conducted using Mplus 8.3 to evaluate the discriminant validity of the five latent variables: digital leadership, work gamification, workplace mindfulness, AI anxiety, and resilience. As shown in Table 2, the five-factor model provided the best fit (χ^2^/df = 1.832, RMSEA = 0.053, CFI = 0.907, TLI = 0.902, SRMR = 0.058), indicating satisfactory discriminant validity.

### 4.2. Common Method Bias Test

Harman’s single-factor test was conducted using SPSS 26. The first unrotated principal component explained 39.230% of the total variance, below the 40% threshold, suggesting that common method bias was within acceptable limits. Additionally, a common method factor was introduced in Mplus version 8.3. As shown in Table 3, the changes in fit indices between the five-factor model and the model with the common method factor were minimal (ΔRMSEA = 0.001, ΔCFI = 0.009, ΔTLI = 0.005, ΔSRMR = 0.001), with changes in CFI and TLI well below 0.1 and changes in RMSEA and SRMR well below 0.05. These results indicate that the inclusion of a common method factor did not substantially alter the model fit, confirming that common method bias is not a serious concern in the present data.

### 4.3. Descriptive Statistics and Correlation Analysis

The results of the descriptive statistics and bivariate correlation analyses are presented in Table 4. Digital leadership was significantly and positively correlated with employee resilience (r = 0.844, *p* < 0.01), work gamification (r = 0.530, *p* < 0.01), and mindfulness in the workplace (r = 0.308, *p* < 0.01). Work gamification was significantly and positively correlated with employee resilience (r = 0.589, *p* < 0.01), and workplace mindfulness was also significantly and positively correlated with employee resilience (r = 0.386, *p* < 0.01). These results provide preliminary support for our hypotheses. To demonstrate that the correlation was not related to collinearity, this study conducted a multicollinearity diagnosis for each predictor variable. The results show that the VIF ranged from 1.401 to 2.188, which is far less than 5, and the tolerance ranged from 0.457 to 0.714, which is much higher than 0.1. There was no serious multicollinearity problem in the data of this study.

### 4.4. Hypothesis Testing

Direct effect test. The results of the hierarchical regression analysis, with employee resilience as the dependent variable, are presented in Table 5. As shown in Models 2 and 7, digital leadership is directly positively correlated with employee resilience (β = 0.848, *p* < 0.001). Therefore, H1 is supported.

Mediation Analysis. The results of the hierarchical regression analyses with gamification at work and mindfulness in the workplace as dependent variables are presented in Table 6. As shown in Models 2 and 4, digital leadership was significantly positively correlated with work gamification (β = 0.537, *p* < 0.001) and workplace mindfulness (β = 0.314, *p* < 0.001). Therefore, H2 and H4 are supported in this study. As shown in Model 11 of Table 5, work gamification is significantly positively correlated with employee resilience (β = 0.156, *p* < 0.001), whereas the relationship between workplace mindfulness and employee resilience is not statistically significant (β = 0.069, *p* = 0.060). To further examine the mediating effects, this study employed PROCESS Model 4 with 5000 bootstrap samples, reporting the standard errors and 95% confidence intervals of the indirect effects. The results are shown in Table 7. The indirect effect of digital leadership on employee resilience through work gamification was 0.081, with a 95% confidence interval of [0.024, 0.137], which did not include zero. Thus, H3 is supported. The indirect effect of digital leadership on employee resilience through workplace mindfulness was 0.021, with a 95% confidence interval of [0.0001, 0.049], which also did not include zero. Therefore, H5 is supported by the data. Although workplace mindfulness does not appear to be significantly correlated with employee resilience, it was indirectly associated with employee resilience through digital leadership. These findings suggest that the degree of association between workplace mindfulness and employee resilience may depend on certain preconditions or external factors. This study indicates that digital leadership functions as an external environmental factor. As discussed in Section 5.1, it helps employees accumulate and mobilize psychological resources through supportive behaviors, thereby fostering employee resilience.

**Figure 2:**
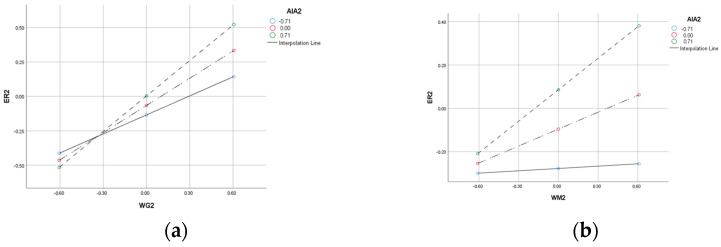
(**a**) Moderating effect of AI anxiety on the relationship between work gamification and employee resilience; (**b**) moderating effect of AI anxiety on the relationship between workplace mindfulness and employee resilience.

Moderation and moderated mediation analyses. The results of the moderation and moderated mediation analyses are reported as follows. As shown in Model 5 and Model 10 of Table 5, the interaction term between work gamification and AI anxiety significantly and positively predicts employee resilience (β = 0.086, *p* < 0.01), and the interaction term between workplace mindfulness and AI anxiety is significantly positively correlated with employee resilience (β = 0.112, *p* < 0.001). These findings indicate that AI anxiety positively moderates the relationships between work gamification and employee resilience as well as between workplace mindfulness and employee resilience. To further examine the moderating role of AI anxiety, this study employed the PROCESS macro (Model 1) with the bootstrap method. The detailed results are presented in Table 8. The findings show that when AI anxiety is low, the 95% confidence interval for the effect of work gamification on employee resilience is [0.325, 0.612], which does not include zero, indicating a significant positive correlation (β = 0.469, *p* < 0.001). When AI anxiety is high, the 95% confidence interval is [0.705, 1.037], which also does not include zero, and the positive correlation remains significant (β = 0.871, *p* < 0.001). As the level of AI anxiety increases, the effect size becomes stronger, suggesting that the relationship between work gamification and employee resilience is positively moderated by AI anxiety. A similar pattern is observed for workplace mindfulness. When AI anxiety is low, the 95% confidence interval is [−0.140, 0.223], which includes zero, indicating that the positive correlation between workplace mindfulness and employee resilience is not significant (β = 0.040, *p* > 0.05). However, when AI anxiety is high, the 95% confidence interval is [0.289, 0.691], which does not include zero, suggesting that workplace mindfulness and employee resilience are significantly positively correlated (β = 0.490, *p* < 0.001). These results indicate that as the level of AI anxiety increases, the positive correlation between workplace mindfulness and employee resilience becomes stronger. This finding aligns with the logic of COR theory: AI anxiety, as a perceived resource threat, prompts employees to mobilize existing resources to enhance their resilience. The specific patterns of these changes are visually presented in Figure 2. Therefore, H6a and H6b are supported.

To examine the moderated mediation effects and the moderating role of AI anxiety on the direct path in the full model, we employed the PROCESS macro (Model 15) with 5000 bootstrap samples. According to Table 9, the interaction between digital leadership and AI anxiety yielded a coefficient of −0.148 (95% CI [−0.229, −0.067], excluding zero), confirming that AI anxiety significantly and negatively moderated the direct relationship between digital leadership and employee resilience, supporting Hypothesis 8. According to Table 10, with respect to the moderated mediation through work gamification, the indirect effects were significant at low, moderate, and high levels of AI anxiety; however, the difference between the high and low groups was not significant (effect = 0.047, 95% CI [−0.003, 0.108], including zero), failing to support Hypothesis 7a. This result may stem from the stability of work gamification. As determined by digital leadership, the institutional design of work gamification requires time for feedback and implementation. As an environmental factor, the enjoyment derived from gamification in the short term is also relatively stable and thus less susceptible to the moderating influence of AI anxiety. With respect to moderated mediation through workplace mindfulness, the indirect effect was nonsignificant for low AI anxiety (effect = −0.007, 95% CI [−0.050, 0.021]) but significant for high AI anxiety (effect = 0.037, 95% CI [0.007, 0.085]), and the difference between the high and low groups was significant (effect = 0.031, 95% CI [0.012, 0.079]), supporting Hypothesis 7b.

### 4.5. Supplementary Experiments

To make the model construction more complete and accurate, this study employed Model 6 to explore chain mediation. The results indicated that the chain mediation path of “digital leadership → work gamification → workplace mindfulness → employee resilience” was not significant, with an effect value of 0.019 and a 95% confidence interval of [−0.001, 0.048]. Since the 95% confidence interval included 0, it was more reasonable to set it as parallel mediation.

## 5. Discussion

### 5.1. General Discussion

Drawing on social cognitive theory, this study investigates the relationship between digital leadership and employee resilience. By examining the reciprocal relationships among environmental, personal, and behavioral factors, we tested the direct effect of digital leadership on employee resilience; the mediating roles of work gamification and workplace mindfulness; and the moderating role of AI anxiety across three pathways. The results revealed that digital leadership not only has a positive correlation with employee resilience but also indirectly promotes it through work gamification and workplace mindfulness, suggesting that digital leadership is an empowerment function that operates through both environmental and cognitive channels. Furthermore, higher levels of employee AI anxiety attenuated the direct positive relationship between digital leadership and employee resilience. However, from the perspective of the personal cognition pathway, higher AI coupled with stronger digital leadership is associated with greater employee mindfulness. These findings collectively illustrate the mutual interactions among the organizational environment, employee cognition, and employee behavior.

Notably, the moderating effect of AI anxiety on the institutional resource pathway was not significant; that is, variations in AI anxiety did not alter the strength of the indirect relationship between digital leadership and employee resilience through work gamification, failing to support Hypothesis 7a. A plausible explanation is as follows: First, in terms of the degree of perfection of work gamification, when leaders possess strong digital leadership, they tend to introduce gamification elements that match the organization’s culture and the overall characteristics of its workforce. A well-designed gamified work system enables employees to rapidly integrate into a positive work atmosphere, maintaining a transcendent positive psychological state within a “levelling up” institutional environment. When employees maintain a positive and upwards-oriented work mentality, they develop a robust capacity for stress management that preserves their learning ability and enhances their occupational health resilience. The entire pathway—from technological environmental change through institutional reform to behavioral influence—is inherently stable. Consequently, this pathway is relatively unaffected by AI anxiety, rendering the moderating effect negligible. Second, the specificity of the AI anxiety measurement is limited. In this study, the scale for AI anxiety is oriented toward the two dimensions of “AI learning anxiety” and “job replacement anxiety”, which are from the perspective of employees’ psychology. However, digital leadership is a manifestation of a leader’s management ability, and work gamification is a work system that involves the task design of an enterprise. Its impact on employee resilience is a kind of interesting experience that is quite different from the psychological states of job replacement anxiety and learning anxiety. The content of the AI anxiety scale does not involve aspects related to gamification and leadership; thus, the path through which AI anxiety is regulated through work gamification as a mediating variable is not significant. Finally, from the perspective of the data collection method, the data collected in this study are cross-sectional data. The moderating effect of AI anxiety on the path mediated by work gamification has a lag. It may be that the erosion of positive experiences by AI anxiety is slow, which is a gradual process. It is also possible that although AI anxiety is high, under the digital leadership of enterprise managers, improving the work gamification system in the enterprise takes time, and an improved gamified work environment may be more conducive to enhancing employee resilience. Therefore, this path should be explored at multiple time points.

Furthermore, in this study, although workplace mindfulness does not directly affect employee resilience, it can act as a mediating variable between digital leadership and employee resilience, exerting an indirect influence on employee resilience. It is also associated with employee resilience under the moderating effect of AI. A reasonable explanation is as follows: According to the conservation of resources theory, workplace mindfulness is a psychological resource, and employee resilience can be regarded as the behavioral outcome of resource accumulation or depletion, as well as an adaptive result to the environment. According to the acquisition of resources in the conservation of resources theory, employees will mobilize their own resources for their own sense of security and desire to continuously accumulate and obtain new psychological resources. Therefore, under the impact of AI anxiety, employees constantly use their own mindfulness resources to change their resilience and utilize external resources to change their level of mindfulness. However, the mobilization of mindfulness levels requires conditions, and it is difficult for it to increase or decrease by itself; thus, it is difficult for mindfulness to directly affect employee resilience. However, when under the management of leaders with strong digital capabilities, enterprises may introduce digital technologies to enhance employees’ mindfulness. To obtain more personal resources, employees accept digital resources within the enterprise and convert enterprise resources into their own psychological resources, thereby enhancing their mindfulness, improving their adaptability, and increasing their resilience. Similarly, mindfulness is an uncritical acceptance of the present and does not have a clear action orientation. However, employee resilience is a behavioral outcome; therefore, it is difficult for mindfulness itself to be significantly associated with employee resilience.

### 5.2. Theoretical Contributions

First, this study expands the research perspective of digital leadership from the macro level to the micro level. Leaders’ mastery and understanding of digital technology affect not only organizational operational efficiency but also employees’ occupational health and psychological well-being. However, prior research has focused predominantly on the impact of digital technology on macrolevel organizational performance. For example, how digital leadership enhances organizational innovation performance (Benitez et al., 2022), while rarely examining the effects of digital technology on individual employees’ occupational health from a microlevel perspective (Xu et al., 2025). By adopting an occupational health psychology framework, this study addresses the scholarly interest in understanding the mechanisms underlying employee mental health outcomes in digitalized contexts, and shifts the analytical focus of digital leadership from organizational performance to employees’ psychological resources.

Second, this paper combines social cognitive theory with COR theory, integrating individual and environmental factors into a unified framework, and expands the multiple paths through which digital leadership influences employee resilience. Existing studies have tended to investigate individual (e.g., proactive personality) and environmental (e.g., high-performance work systems) factors in isolation (Cooke et al., 2016; Jeong & Jeong, 2025; Luan et al., 2025) without integrating them within a unified framework. This study incorporates the digital technology environment into the research design and examines not only the direct effect of digital leadership on employee resilience but also the indirect effects through two pathways: work gamification (environment → behavior) and workplace mindfulness (environment → cognition → behavior). This study also analyzed the reasons for the existence of the moderated mediation path and the direct path from the perspective of COR theory. These findings validate the applicability of social cognitive theory and COR theory in the occupational health domain.

Third, this paper, based on employee’s cognitive factors, incorporates AI anxiety into the research framework, thereby expanding the boundary conditions of the mechanism through which employee resilience exerts its influence. Prior research has typically examined employee resilience within conventional business environments (Hallak et al., 2018) or sudden crisis contexts (Luan et al., 2025), often without quantifying the severity of environmental shocks, making it difficult to precisely capture the psychological impact of environmental change. Against the backdrop of AI’s rapid penetration into the workplace, this study quantifies employees’ negative psychological responses to AI as AI anxiety, capturing the impact of technological change on employees’ occupational health at the cognitive level. The finding that AI anxiety both negatively moderates the direct effect of digital leadership on employee resilience and positively moderates the mediating pathway through workplace mindfulness reveals the dual role of technological anxiety in the relationship between organizational resources and employee health: it can weaken the protective function of leadership while simultaneously activating employees’ internal psychological resources. These findings provide new empirical support for “stressor–resource” interaction theory in the domain of occupational health and provides a referable analytical framework for future research on the relationship between technostress and psychological resilience.

### 5.3. Practical Implications

First, when seeking to enhance employee resilience, organizational leaders should proactively monitor digital technology development and introduce the necessary digital resources with effective implementation strategies. Digital leadership is not merely a reflection of technological competency but also a critical management resource for safeguarding employees’ psychological health. Leaders should strengthen their understanding and application of digital technology, guide employees in using digital tools appropriately, and mitigate the occupational stress and anxiety caused by technological unfamiliarity or misuse. Specifically, organizations can regularly organize digital technology workshops to identify internal technology gaps and develop solutions while providing middle managers with digital competency training to ensure that they serve as effective bridges between senior leadership strategy and employee execution.

Second, management teams should systematically monitor the application of digital resources and actively promote the implementation of gamification at work and mindfulness training for employees. Gamified work systems can transform repetitive tasks into intrinsically motivating activities, helping employees gain a sense of accomplishment and control within a “leveling up” institutional framework, thereby alleviating work stress and strengthening psychological resilience. Organizations may consider introducing gamification platforms that enable employees to track their work progress in real time and receive positive feedback. In parallel, organizations can leverage digital technology, such as mobile applications, to conduct regular mindfulness exercises that help employees cultivate attentional focus and emotion regulation capacity. These interventions have been empirically validated as effective in occupational health strategies. By combining institutional design with technological empowerment, organizations can provide employees with sustained psychological support.

Third, organizational leaders should strengthen their humanistic care and pay close attention to the negative impact of AI anxiety on employees’ mental health. During digital transformation, employees’ concerns about technological displacement and skill obsolescence represent real and pervasive occupational stressors. Management should acknowledge the existence of AI anxiety and prioritize it within occupational health management rather than simply attributing it to individual adaptation problems. Different intervention strategies should be adopted for employees with varying levels of anxiety. For those with high AI anxiety, mindfulness training can strengthen internal psychological resources and help maintain focus and stability in uncertain environments. Simultaneously, gamified work design can serve as an emotional buffer, helping employees resolve resistance to technology through positive experiences. Through these measures, organizations can enhance employee resilience while building a more compassionate organizational culture amid technological change, effectively preventing burnout and psychological problems.

### 5.4. Limitations and Future Directions

First, to better investigate employee resilience, this study conducted questionnaire surveys on employees from different regions through two channels. Harman’s single-factor test was also carried out, and a common method factor was introduced, but common method bias was still difficult to eliminate. Although employees can objectively rate their own mindfulness levels, occupational health resilience capabilities, and AI anxiety, they may have limited insight into management’s digital leadership or the progress of work gamification implementation. Relying solely on employee-reported data may introduce bias. Future research should simultaneously survey both employees and leaders or employ a leader–employee matched design (e.g., one leader matched with three employees). This approach provides more objective assessments of whether managers’ digital leadership genuinely achieves the goal of enhancing employee resilience and whether the gamification elements being implemented are universally beneficial for employee capacity building. Additionally, collecting data from managers at different hierarchical levels could enable a deeper investigation of whether mindfulness training and gamification implementation are hindered by oversight at any particular managerial level. Similarly, as this collection is cross-sectional data, it is impossible to completely rule out the potential impact of common method bias. In the future, longitudinal data can be collected for tracking, attention test questions can be added to the questionnaire design, or different scoring methods can be adopted for different variables to increase the validity of the data.

Second, this study revealed that workplace mindfulness did not directly predict employee resilience; however, when workplace mindfulness served as a mediator between digital leadership and employee resilience, the mediation pathway was significant. When the moderating effect of AI anxiety was considered, employee mindfulness was positively correlated with employee resilience. The framework for the relationship between workplace mindfulness and employee resilience in this study is still not well established. Future research should further explore the conditions under which workplace mindfulness affects employee resilience. Moreover, as both mindfulness and employee resilience are core pillars of positive psychology, when the relationship between workplace mindfulness and employee resilience is studied in the future, positive psychology can be introduced to construct relevant models to explore the relationship between the two and provide more effective microlevel suggestions on how to enhance employee resilience. Third, this study explored only the relationship between digital leadership and employee resilience from the perspective of “environment → individual → behavior” on the basis of a single path of social cognitive theory. However, investigations of the possible relationships among work gamification, workplace mindfulness, and employee resilience based on the triadic interaction foundation of social cognitive theory are lacking. Moreover, owing to the limitations of cross-sectional data, this study failed to explore in depth the “individual behavior → environment” path; that is, whether employee resilience affects work gamification and digital leadership inversely, nor does it delve into the “behavior → individual → environment” path and whether employee resilience inversely affects the level of mindfulness to promote changes in digital leadership. Future research could adopt panel data for long-term tracking to obtain more accurate research data and analyse whether there are potential mediating paths between work gamification and workplace mindfulness, as well as the relationships between employee resilience and individual and environmental factors.

Finally, the measurement of AI anxiety in this study has several limitations. This study selected AI anxiety, which includes AI learning anxiety and job replacement anxiety. However, subtle differences may exist between them. Future research should select two different scales for AI learning anxiety and job replacement anxiety to analyse the relationships between different dimensions of AI anxiety and different variables from a more microscopic perspective, making the research results more accurate.

## Figures and Tables

**Table 1:** Sample characteristics.

Category	Feature	Proportion
Gender	Male	53.6%
	Female	46.4%
Age	Under 30	23.9%
	30–45 (inclusive)	46.1%
	45–55 (inclusive)	21.5%
	Over 55	8.5%
Education	Associate degree or below	17.7%
	Bachelor’s degree	48.8%
	Master’s degree	23.9%
	Doctoral degree or above	9.6%
Company size	50 or fewer	19.5%
	51–300	37.5%
	301–500	23.5%
	Over 500	19.5%
Company type	State-owned	22.5%
	Private	39.6%
	Foreign-invested	18.8%
	Collective	9.2%
	Other	9.9%
Region	Eastern China	42.3%
	Central China	20.5%
	Western China	29.7%
	Northeast China	7.5%

**Table 2:** Confirmatory factor analysis results.

Model	χ^2^	df	χ^2^/df	RMSEA	CFI	TLI	SRMR
Five-factor: DL, ER, AIA, WG, WM	2212.968	1208	1.832	0.053	0.907	0.902	0.058
Four-factor: DL + ER, AIA, WG, WM	2342.602	1196	1.959	0.057	0.894	0.887	0.048
Four-factor: DL, ER, AIA, WG + WM	2396.671	1212	1.977	0.058	0.890	0.885	0.104
Four-factor: DL, ER, AIA + WG, WM	3282.996	1209	2.715	0.077	0.808	0.798	0.090
Three-factor: DL + ER + AIA, WG, WM	3073.266	1203	2.555	0.073	0.827	0.817	0.115
Two-factor: DL + ER + AIA + WG, WM	4297.601	1218	3.529	0.093	0.715	0.702	0.123
One-factor: DL + ER + AIA + WG + WM	7272.645	1224	5.942	0.130	0.441	0.417	0.150

Note: DL = digital leadership; WG = work gamification; WM = workplace mindfulness; ER = employee resilience; AIA = AI anxiety.

**Table 3:** Model fit after adding the common method factor.

Model	χ^2^	df	χ^2^/df	RMSEA	CFI	TLI	SRMR
Five-factor: DL, ER, AIA, WG, WM	2212.968	1208	1.832	0.053	0.907	0.902	0.058
Five-factor + CMV	2069.474	1158	1.787	0.052	0.916	0.907	0.057
Difference	143.493	50	0.045	0.001	0.009	0.005	0.001

Note: DL = digital leadership; WG = work gamification; WM = workplace mindfulness; ER = employee resilience; AIA = AI anxiety.

**Table 4:** Descriptive statistics and correlation matrix.

Variable	M	SD	1	2	3	4	5
1. DL	4.040	0.750	(0.931)				
2. ER	3.998	0.724	0.844 **	(0.948)			
3. WG	3.810	0.605	0.530 **	0.589 **	(0.881)		
4. AIA	3.437	0.712	0.349 **	0.422 **	0.549 **	(0.886)	
5. WM	3.611	0.609	0.308 **	0.386 **	0.565 **	0.705 **	(0.926)

Note. *N* = 293. ** *p* < 0.01. The values in parentheses are the Cronbach’s α coefficients. Control variables (gender, age, education, company size, company type, and region) are omitted for brevity. Note: DL = Digital leadership; WG = Work gamification; WM = Workplace Mindfulness; ER = Employee Resilience; AIA = AI Anxiety.

**Table 5:** Results of the Hierarchical Regression Test (with Employee Resilience as the result Variable).

Variables	Work Gamification					Workplace Mindfulness					Overall		
	Model 1	Model 2	Model 3	Model 4	Model 5	Model 6	Model 7	Model 8	Model 9	Model 10	Model 11	Model 12	Model 13
Control variables													
Gender	0.008	0.030	0.020	0.021	0.021	0.008	0.030	0.030	0.029	0.029	0.022	0.022	0.020
Age	−0.047	−0.014	−0.012	−0.010	−0.006	−0.047	−0.014	−0.020	−0.014	−0.016	−0.016	−0.012	−0.021
Education	−0.040	0.007	0.007	0.014	0.012	−0.040	0.007	0.004	0.013	0.013	0.005	0.012	0.003
Enterprise size	−0.033	−0.023	−0.002	−0.001	0.007	−0.033	−0.023	−0.013	−0.011	−0.014	−0.002	−0.001	0.000
Enterprise nature	0.019	−0.039	−0.030	−0.027	−0.030	0.019	−0.039	−0.035	−0.032	−0.028	−0.029	−0.028	−0.031
Enterprise area	−0.026	0.006	−0.005	−0.007	−0.009	−0.026	0.006	0.004	0.001	0.002	−0.004	−0.006	−0.008
IV													
Digital Leadership (DL)		0.848 ***	0.743 ***	0.737 ***	0.715 ***		0.848 ***	0.805 ***	0.792 ***	0.767 ***	0.742 ***	0.738 ***	0.670 ***
Mediator													
Work gamification (WG)			0.194 ***	0.153 ***	0.180 ***						0.156 ***	0.145 **	0.183 ***
Workplace mindfulness (WM)								0.137 ***	0.077	0.099 *	0.069	0.032	0.050
Moderator													
AI anxiety				0.082 *	0.065				0.091 *	0.077		0.063	0.075
IV × Mediator													
DL × AI anxiety													−0.123 ***
WG × AI anxiety					0.086 **								0.084 **
WM × AI anxiety										0.112 ***			0.100 *
R^2^	0.005	0.715	0.742	0.746	0.753	0.005	0.715	0.732	0.736	0.748	0.745	0.747	0.770
ΔR^2^	0.005	0.710	0.026	0.005	0.007	0.005	0.710	0.017	0.004	0.012	0.030	0.002	0.023
F	0.257	710.819 ***	28.974 ***	5.095 *	7.583 **	0.257	710.819 ***	17.824 ***	4.181 *	13.222 ***	16.407 ***	2.008	9.264 ***

Note. * *p* < 0.05, ** *p* < 0.01, *** *p* < 0.001.

**Table 6:** Results of hierarchical regression tests (with work gamification and workplace mindfulness as the result variables).

Variables	WG		WM	
	Model 1	Model 1	Model 1	Model 1
**Control variables**				
Gender	0.037	0.051	−0.006	0.002
Age	−0.027	−0.006	0.031	0.044
Education	−0.030	0.000	0.004	0.022
Enterprise size	−0.113	−0.107	−0.073	−0.069
Enterprise nature	−0.013	−0.050	−0.009	−0.031
Enterprise area	0.040	0.061	0.003	0.015
**IV**				
Digital Leadership (DL)		0.537 ***		0.314 ***
R^2^	0.018	0.302	0.007	0.104
ΔR^2^	0.018	0.285	0.007	0.097
F	0.854	116.309 ***	0.340	30.932 ***

Note: DL = digital leadership; WG = work gamification; WM = workplace mindfulness. *** *p* < 0.001.

**Table 7:** Mediation effect results (bootstrap = 5000).

Pathway	Effect	SE	95% CI Lower	95% CI Upper
Total effect	0.818	0.031	0.757	0.878
Total indirect effect	0.102	0.028	0.050	0.159
DL → WG → ER	0.081	0.029	0.024	0.137
DL → WM → ER	0.021	0.012	0.0001	0.049

Note: DL = digital leadership; WG = work gamification; WM = workplace mindfulness; ER = employee resilience.

**Table 8:** Results of Moderating Effects.

Variables	Moderator	Effect Size	SE	*p* Value	95% CI
Interaction term (WG × AIA)	-	0.282	0.060	0.000	[0.164, 0.400]
Conditional effect (WG → ER)	Low AI	0.469	0.073	0.000	[0.325, 0.612]
	Moderate AI	0.670	0.066	0.000	[0.539, 0.800]
	High AI	0.871	0.084	0.000	[0.705, 1.037]
Interaction term (WM × AIA)	-	0.316	0.063	0.000	[0.193, 0.439]
Conditional effect (WM → ER)	Low AI	0.040	0.093	0.664	[−0.140, 0.223]
	Moderate AI	0.265	0.087	0.003	[0.094, 0.437]
	High AI	0.490	0.102	0.000	[0.289, 0.691]

Note: WG = work gamification; WM = workplace mindfulness; ER = employee resilience; AIA = AI anxiety.

**Table 9:** The Moderating Effect of AI Anxiety between Digital Leadership and Employee Resilience.

Variables	Moderator	Effect Size	SE	*p* Value	95% CI
Interaction term (DL × AIA)	-	−0.148	0.041	0.000	[−0.229, −0.067]
Conditional effect (DL → ER)	Low AI	0.751	0.039	0.000	[0.675, 0.828]
	Moderate AI	0.646	0.036	0.000	[0.576, 0.717]
	High AI	0.541	0.052	0.000	[0.438, 0.644]

Note: DL = digital leadership; ER = employee resilience; AIA = AI anxiety.

**Table 10:** Moderated mediation effects (bootstrap = 5000).

Mediator	AI Anxiety Level	Indirect Effect	SE	95% CI
Work gamification	Low (M − 1SD)	0.062	0.029	[0.001, 0.119]
	Moderate (M)	0.095	0.025	[0.047, 0.142]
	High (M + 1SD)	0.128	0.033	[0.065, 0.194]
	High − Low difference	0.047	0.026	[−0.003, 0.108]
Workplace mindfulness	Low (M − 1SD)	−0.007	0.018	[−0.050, 0.021]
	Moderate (M)	0.015	0.015	[−0.012, 0.046]
	High (M + 1SD)	0.037	0.020	[0.007, 0.085]
	High − Low difference	0.031	0.017	[0.012, 0.079]

## Data Availability

The data supporting the findings of this study are available from the corresponding authors upon reasonable request.

## Abbreviations

The following abbreviations are used in this manuscript:

DL	Digital Leadership
WG	Work Gamification
WM	Workplace Mindfulness
AIA	AI Anxiety
ER	Employee resilience
CFA	Confirmatory Factor Analysis
CMV	Common Method Variance
